# Use of Digital Panoramic Radiographs in the Study of Styloid Process Elongation

**DOI:** 10.1155/2015/474615

**Published:** 2015-07-28

**Authors:** Carla Cabral dos Santos Accioly Lins, Renan Macêdo Cutrim Tavares, Camila Caroline da Silva

**Affiliations:** ^1^Department of Anatomy, Federal University of Pernambuco (UFPE), Avenida Professor Moraes Rego S/N, Cidade Universitária, 50670-901 Recife, PE, Brazil; ^2^Department of Prosthodontics and Oral Facial Surgery, School of Dentistry, Federal University of Pernambuco (UFPE), Avenida Professor Moraes Rego S/N, Cidade Universitária, 50670-901 Recife, PE, Brazil

## Abstract

This work aimed to evaluate the occurrence of suggestive images of styloid process elongation in panoramic radiographs, noting their frequency according to sex, age, and location, as well as measure and classify the types and patterns of calcification of elongated styloid processes. 2,500 panoramic radiographs were evaluated in a Radiology Clinic in Recife, PE, Brazil, performed between 2008 and 2010, with the age ranging from 25 to 80 years old. 560 of the radiographs analyzed fulfilled the inclusion criteria. Of this total, 216 (38.57%) presented suggestive images of the styloid process elongation, 45 (20.8%) belonging to male and 171 (79.2%) to female, and 84.7% were bilateral. After all measurements, mean values of 35.5 mm (left side) and 37.6 mm (right side) were obtained and these differences were statistically significant (*p* < 0.001). The most common type of stretching found was elongated (type I) with 73.1%, and the pattern of calcification was partially calcified (62.5%). It was found that the elongation of the styloid process is an anatomical variation, which must be taken into account by dentists, and because panoramic radiography is a technique of easy approach and low cost and routine, it can be used to aid in the diagnosis of elongated styloid process.

## 1. Introduction

The styloid process (SP) is a slender, pointed bony structure that protrudes downward and forward from the lower surface of the temporal bone, anteromedially to the stylomastoid foramen. It is located between the internal and external carotid arteries, after the pharynx, giving rise to stylohyoid, styloglossus, and stylopharyngeus muscles and stylohyoid and stylomandibular ligaments [1, 2]. Its embryonic origin is in the Reichert cartilage of the second branchial arch and, along with the stylohyoid ligament and the lesser horn of the hyoid bone, forms the stylohyoid complex [3].

The elongation of the SP is an anomaly that may be accompanied by calcification of stylohyoid and stylomandibular ligaments, potentially triggering a series of symptoms, such as foreign body sensation in the throat, pain when moving the head, vertigo, dysphagia, odynophagia, facial pain, earache, headache, tinnitus, and trismus. This set of symptoms associated with elongated styloid process is called Eagle's syndrome (ES) [4].

The analysis of radiographs and clinical examination of the patient are considered important tools to confirm the diagnosis of elongated styloid process (ESP) and Eagle's syndrome. Care must be taken to make the differential diagnosis with the dysfunctions of temporomandibular joint, tumors of tongue base, and trigeminal and glossopharyngeal neuralgia [5], as well as migraine states, unerupted third molars, myofascial pain, and cervical arthritis [6].

Thus, this study aimed to identify the prevalence of elongated styloid process in digital panoramic radiographs, aiming to evaluate the occurrence regarding sex and location (unilateral or bilateral), as well as measure the length of the elongated styloid process and classify the pattern of calcification and the type of elongation.

## 2. Materials and Methods

This study was approved by the Ethics Committee on Human Research of Federal University of Pernambuco (UFPE), Brazil (CAAE number: 0195.0.172.000-11). The material used was composed of 2,500 digital panoramic radiographs obtained from 2008 to 2010, belonging to the archive from a Radiology Clinic of Recife, PE, Brazil. Inclusion criteria were age between 25 and 80 years, both sexes, and X-rays that allow the visualization of the start and end points of the styloid process from both sides.

**Figure 1:**
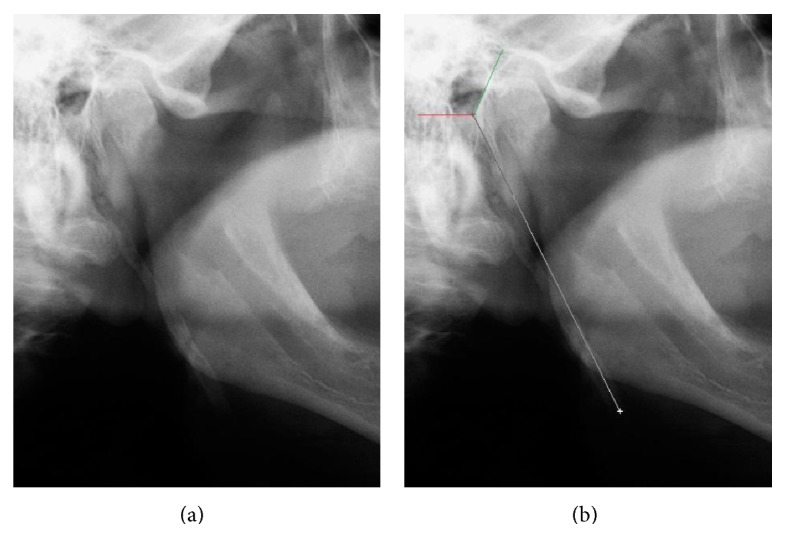
(a) Panoramic radiography showing the elongated styloid process. (b) Measurement of the styloid process using the software's rule (the red line shows the lower limit of the external ear canal and the green the previous limit).

A single trained individual, using HP G60 notebook with a 17-inch screen in a dark room for better viewing, evaluated the images. Radiographs in which the styloid exceeded 30 mm from the lower edge were considered suggestive of elongation. The initial reference point used for the measurement was the lower edge of the ear canal and the end point of the end of the styloid process. The measurements were made with the ruler of the image manipulation software Adobe Photoshop CS3 (with magnification and manipulation of brightness and contrast features) that was positioned on the radiographs and displayed on the monitor (Figure 1).

Each device possessed a distortion previously provided by the manufacturer, with the Cranex D equal to 1.34 and 1.27 for the Kodak. Then, a simple ratio calculation was used for the actual size of the styloid processes:

(1) $$\begin{array}{c} RS=\frac{VS}{D}, \end{array}$$

where RS is real size, VS is virtual size, and D is distortion.

The evaluations of the calcification pattern and type of elongation were made by styloid process parameters used by Langlais et al. (1986) [7], who rated the types of elongation: elongated or type I: characterized by a continuous and full mineralization of the complex; pseudoarticulated or type II: in which the styloid process apparently interacts with the stylomandibular and stylohyoid ligaments by a single pseudojoint; and segmented or type III: characterized by a lack of mineralization contiguity of the process or stylohyoid ligament. In this case, the measurement was performed from the initial point to the most distal point, regardless of the distance between the segments. Regarding calcification patterns, the same authors made classification as follows: calcified outline, with a thin edge with a central radiopaque radiolucency which constitutes most of the process; partially calcified, which features a radiopaque thicker layer with small and discontinuous radiolucent centers; nodular, which presents as calcified nodes and can be partially or completely calcified with various degrees of calcification; and calcified pattern that is totally calcified without evidence of radiolucent area in its interior.

Data regarding age, sex, right and left sides, length, type and pattern of calcification, and apparatus used were transferred to a spreadsheet in Microsoft Excel 2007; and the statistical calculations were obtained by using SPSS (Statistical Package for Social Sciences) version 21 software. The margin of error used in the decision of the statistical tests was 5.0%.

For data analysis, the absolute distribution, percentages, and statistics measures were obtained: mean, standard deviation, median, and minimum and maximum values (descriptive statistical techniques) and the following statistical tests were used: Wilcoxon test for paired data, McNemar Bowker, Kruskal-Wallis with multiple comparisons in case of significant differences, Mann-Whitney, and Chi-square or Fisher's exact test when the conditions for the Chi-square test were not verified (inferential statistical techniques).

## 3. Results

Of the 2,500 radiographs analyzed, 560 matched the inclusion criteria, and of this total, 216 presented the elongated SP. Of these patients, 93 (43.1%) were between 25 and 39 years old, 93 (43.1%) were between 40 and 59 years old, and 30 (13.9%) were 60 to 80 years old; and 45 (20.8%) were male and 171 (79.2%) were female.

Table 1 shows the results of the length of the SP, the pattern of mineralization, and type of elongation. From this table it is emphasized that the length means ranged from 35.5 mm (left side) to 37.6 mm (right side), and these differences were statistically significant (*p* < 0.001). On both sides, the partially calcified pattern was the most prevalent, with 62.5%, and elongated, with 75.0% on the right side and 71.3% on the left side, but the margin of error fixed (5.0%) did not show significant differences between the sides in relation to the pattern of calcification and elongation type (*p* > 0.05).

The majority of patients had bilateral elongation (84.7%), followed by 13.0% unilateral left and right side by 2.3%. Tables 2 and 3 show the statistics of the length of the SP according to the pattern of mineralization and type of elongation. It was verified, on each side, that the mean length was correspondingly higher among patients with calcified pattern and lower among calcified outline patients; however, there were no significant differences between the patterns of calcification on either side (*p* > 0.05).

Table 3 shows that, on each side, the mean length was correspondingly higher among patients with pseudoarticulated type of elongation. On the right side, the lower average was observed in elongated type and the left side between segmented cases, proving significant difference between the types of elongation on the left side (*p* < 0.001) and by multiple comparison tests (paired) a significant difference is demonstrated between each pair of types.

Table 4 presents the results of the length of SP according to each of the variables: age and sex. A significant difference between sexes was verified on the left side, with the highest mean in males compared to females (38.3 × 34.8). Means were correspondingly higher on the right side than on the left, and with the exception of males, we see a significant difference between the sides in the other situations (*p* < 0.05). When the pattern of calcification in each side for age and sex was analyzed, no significant associations between each variable and calcification pattern were observed both on the right side and on the left side (*p* > 0.05), as presented results in Tables 5 and 6.

Tables 7 and 8 examined the types of elongation on each side by age and sex of patients. There was a significant association between age and type of elongation on the right side, and for this variable it is emphasized that the percentage of elongated type increased with age, with 64.5% between 25 and 39 years old and 86.7% between 60 and 80 years old, while the percentage of segmented type was higher in the range of 25–39 years (32.3%) and ranged from 12.9% to 13.3% in the other two age groups.

Significant association between age and type of elongation on the left side is verified in Table 8, and for this variable it stands out that the percentage of the elongated type was lower in the age group of 25–39 years old (59.1%) and ranged from 80% to 80.6% in the other two age groups, while the percentage of segmented type was higher in the range of 25–39 years old (37.6%) and ranged from 14.0% to 20.0% in the other two age groups (*p* < 0.05).

After the completion of the association between the type of elongation and pattern of calcification by side, on the right side (Table 9), the significant association between the two variables was observed (*p* < 0.05), which highlights that the largest percentage differences occurred between segmented type and elongated with calcified outline type of elongation, with highest in the segmented type (39.1% versus 22.2%) and among the types: elongated and pseudoarticulated in the calcified outline pattern, with higher value in elongated type (64.2% versus 50.0%).

On the left side (Table 10) there was a significant association between the two variables (*p* < 0.05), which highlights the fact that the largest percentage differences occurred between the elongated and pseudoarticulated types in partially calcified pattern with higher value between the elongated types (64.9% versus 37.5%) and between segmented elongated types in cases with calcified outline, higher on the segmented type (40.7% versus 22.1%).

## 4. Discussion

The incidence of elongated styloid process has a great variability in the population [8]. Studies have shown that its occurrence in panoramic radiographs varies between 4% and 28% [9–12], whereas only 4% to 10.3% of that group are symptomatic [11]. In this work, the observed incidence was 38.57%, differing from other radiologic studies reported in the literature that related 84.4% [13], 32.40% [14], and 29.6% [15].

The use of panoramic radiographs is considered by some authors as an important diagnostic tool for the elongation of the SP [9–12, 14–21]. It is described as a preferred method of choice for this observation, because it is a simple technique of routine use, when overview of the maxillofacial complex is needed, and therefore widely used for this purpose [11]. However, precautions must be taken when conducting and analyzing the images, taking care to stabilize the degree of distortion inherent to technique and equipment. This criterion is very important, since the measurements are milimetrics and numerical changes observed can mean different statistical results.

The methods of measurement of ESP described in the literature are quite varied. Some authors have reported the use of manual calipers and rulers directly on the radiographs [9, 14, 19, 22], while others used planigraphy and digital calipers [20]. Guimarães et al. [11] described that they used as reference the lower edge of the cartilage of the ear lobe, and ESPs which exceeded at least 1 cm were considered elongated; and the method described by More and Asrani (2010) [12] using a software that measured the ESPs, shortly after its emergence from the tympanic plate. In this study the method used was similar to those authors, and to avoid distortion, we used a simple ratio calculation to obtain the actual size.

The ESP's standard size is described ranging from 25 to 30 mm, and when larger it is considered elongated [23, 24]. However, among the surveyed authors, the maximum normal size used as a reference in their studies was 25 mm [11, 13, 15], different from this study, which used as normal maximum size 30 mm, collaborating with other research that applied this same parameter [9, 12, 14, 19–22, 25, 26].

After the measurements, the mean size of the elongated ESPs was 37.6 mm for the right side and 35.5 mm on the left side, agreeing with the findings of Ilgüy et al. [9] who evaluated 860 radiographs, in which 32 presented images suggesting elongation of ESP, and obtained the mean size of 34 and 35 mm in the left and right sides, respectively; data different from the study of Correll et al. [25] who analyzed 1.171 radiographs, and 606 of those presented images suggesting ESP with medium size observed on both sides of 43.6 mm.

Tavares and Freitas [14] evaluated 463 panoramic radiographs, in which 150 of them showed elongation of the SP, and observed that bilateral occurrence was more common (60%) and no significant difference between sexes was observed, with a higher frequency in the age group between 41 and 60 years old. Guimarães et al. [11], analyzing 2,600 X-rays, recorded 146 images of suggestive elongation of the ESP, most commonly found in females in the age of 11–40 years with bilateral occurrence of 84%. In this study, it was observed that the elongation was more present bilaterally (84.7%), resembling this and other studies that showed similar results: 85.75% [25], 71.5% [21], and 67.7% [13].

Regarding the frequency in relation to sex, it was observed to be higher in females with 79.2%, collaborating with studies by Guimarães et al. [11] and de Paula and Carraretto [21] who observed 63% and 86%, respectively. Further studies reported that the frequency in relation to sex is irrelevant [13, 14, 22]. Regarding the age, it was observed that there is no consensus among the authors, because some reported a higher incidence in the age group between 41 and 50 years [10] and the other between 30 and 40 years [24], and others have mentioned a greater increase after 50 years of age [25]. In this research the most affected age groups were between 25 and 39 years old and 40 and 59 years old with equal frequencies of 43.1%.

In this study, the most common type of elongation observed was elongated with 73.1% of patients. Other authors also reported in their studies that the elongated type was the most frequent, however, in varying percentages: 85% [12], 74.2% [21], 71.1% [9], and 68% [10].

Another finding related was the correlation between the type of elongation and age, where it was observed that with increasing age the elongated type of the right side is more often found. However, it was not possible to correlate this information with the literature, since no similar result was found.

When the pattern of calcification was analyzed, it was found that the partially calcified pattern was the most frequent in 62.5% of the radiographs, collaborating with the findings of More and Asrani [12] who evaluated 500 radiographs and observed that 70% of them showed the same pattern and İlgüy et al. [4], who evaluated 860 patients in which 32 showed suggestive images of elongation of the SP, and of these 27 cases showed a partially calcified pattern.

When evaluating how much the length varies on the type and pattern of calcification, it was observed that the calcified pattern and pseudoarticulated type have the greatest lengths of ESP, where the calcified pattern obtained a mean size of 40.5 mm on the right side and 37.7 mm on the left side, and the pseudoarticulated type obtained mean size of 43.4 mm and 52.2 mm on right and the left, respectively. Another new parameter obtained in this study was the relationship between the type of elongation and pattern of calcification, where it was possible to observe that on the right side the partially calcified pattern was more frequently found in all types of elongation (64.2% elongated, 50% pseudoarticulated, and 58.7% segmented). On the left side, the partially calcified pattern was found more frequently in elongated (64.9%) and segmented (59.3%) types, while the pseudoarticulated type and the calcified and partially calcified patterns were each found in 37.5% of cases.

## 5. Conclusion

It was found that the elongation of the styloid process is an anatomical variation, which must be taken into account by dentists; the panoramic radiography as a technique of easy approach and low cost can be used to aid in diagnosis; despite the fact that there are several studies in the literature regarding this topic, a better understanding of the correlations between the types and patterns of calcification should be investigated in daily clinics, in order to associate the presence of styloid process elongation with the clinical symptoms.

## Figures and Tables

**Table 1:** Evaluation of the length pattern of calcification and elongation according to the international side.

Variable	Right	Left	*p* values
Elongation variant: process mean ± SD (median) mm	37.6 ± 7.3 (35.8)	35.5 ± 8.0 (34.4)	*p*(1) < 0.001^*∗*^
Calcification pattern: *n* (%)			
Calcified	24 (11.1)	23 (10.6)	*p*(2) = 0.824
Almost calcified	135 (62.5)	135 (62.5)	
Calcified outline	57 (26.4)	58 (26.9)	
Type of elongation: *n* (%)			
Elongated	162 (75.0)	154 (71.3)	*p*(2) = 0.443
Pseudoarticulated	8 (3.7)	8 (3.7)	
Segmented	46 (21.3)	54 (25.0)	

The results obtained were based on the total number of X-rays analyzed: 216.

^*∗*^Significant diference at 5.0%.

(1) Through the Wilcoxon test for paired data.

(2) Through the McNemar Bowker test.

**Table 2:** Length of the styloid processes according to the pattern of calcification.

Pattern of calcification	Length of the styloid process (mm)	
	Right	Left
	Mean ± SD (median)	Mean ± SD (median)
Calcified	40.5 ± 10.8 (36.5)	37.7 ± 9.6 (35.8)
Almost calcified	37.4 ± 7.3 (35.8)	36.0 ± 7.7 (34.4)
Calcified outline	36.8 ± 5.1 (35.5)	33.5 ± 7.6 (34.2)
**p** **value**	**p**(2) = 0.432	**p**(2) = 0.398

(2) Through the Kruskal-Wallis test.

**Table 3:** Length of the styloid processes according to the type of elongation.

Type of elongation	Length of the styloid process (mm)	
	Right	Left
	Mean ± SD (median)	Mean ± SD (median)
Elongated	36.9 ± 5.8 (35.8)	35.6 ± 5.5 (34.4) (A)
Pseudoarticulated	43.4 ± 15.8 (36.2)	52.2 ± 14.3 (48.9) (B)
Segmented	39.1 ± 9.3 (36.8)	33.0 ± 9.6 (33.2) (C)
**p** **value**	**p**(2) = 0.382	**p**(2) < 0.001^**∗**^

^*∗*^Significant diference at 5.0%.

(2) Using the Kruskal-Wallis test comparisons of that test.

**Table 4:** Evaluation of styloid process length according to age and sex.

Variable	Length of the styloid process (mm)		*p* values
	Right	Left	
	Mean ± SD (median)	Mean ± SD (median)	
Age			
25 to 39	37.6 ± 6.7 (36.2)	35.2 ± 7.6 (34.3)	*p*(1) < 0.001^*∗*^
40 to 59	38.2 ± 8.6 (35.9)	36.5 ± 8.9 (34.5)	*p*(1) < 0.001^*∗*^
60 to 80	35.8 ± 4.1 (34.8)	33.7 ± 5.4 (33.2)	*p*(1) = 0.047^*∗*^
**p** **value**	**p**(2) = 0.538	**p**(2) = 0.424	
Sex			
Male	39.2 ± 10.6 (35.1)	38.3 ± 11.3 (35.8)	*p*(1) = 0.260
Female	37.2 ± 6.1 (36.0)	34.8 ± 6.7 (34.2)	*p*(1) < 0.001^*∗*^
**p** **value**	**p**(3) = 0.567	**p**(3) = ****0.025^*∗*^	

^*∗*^Significant difference at 5.0%.

(1) Through the Wilcoxon test for paired data.

(2) Through the Kruskal-Wallis test.

(3) Using the Mann-Whitney test.

**Table 5:** Evaluation of the pattern of calcification on the right side according to age and sex.

Variable	Calcified		Almost calcified		Calcified outline		Total		*p* values
	*n*	%	*n*	%	*n*	%	*n*	%	
**Total**	**24**	**11.1**	**135**	**62.5**	**57**	**26.4**	**216**	**100.0**	
Age									
25 to 39	8	8.6	55	59.1	30	32.3	93	100.0	*p*(1) = 0.402
40 to 59	13	14	61	65.6	19	20.4	93	100.0	
60 to 80	3	10.0	19	63.3	8	26.7	30	100.0	
Sex									
Male	4	8.9	29	64.4	12	26.7	45	100.0	*p*(1) = 0.866
Female	20	11.7	106	62.0	45	26.3	171	100.0	

(1) Through Pearson's Chi-square test.

**Table 6:** Evaluation of the pattern of calcification on the left side according to age and sex.

Variable	Calcified		Almost calcified		Calcified outline		Total		*p* values
	*n*	%	*n*	%	*n*	%	*n*	%	
**Total**	**23**	**10.6 **	**135**	**62.5**	**58**	**26.9**	**216**	**100.0**	
Age									
25 to 39	8	8.6	60	64.5	25	26.9	93	100.0	*p*(1) = 0.796
40 to 59	12	12.9	58	62.4	23	24.7	93	100.0	
60 to 80	3	10.0	17	56.7	10	33.3	30	100.0	
Sex									
Male	4	8.9	28	62.2	13	28.9	45	100.0	*p*(1) = 0.881
Female	19	11.1	107	62.6	45	26.3	171	100.0	

(1) Through Pearson's Chi-square test.

**Table 7:** Evaluation of the type of elongated styloid process on the right side according to age and sex.

	Type of elongation								
	Elongated		Pseudoarticulated		Segmented		Total		*p* values
	*n*	%	*n*	%	*n*	%	*n*	%	
**Total**	**162**	**75**	**8**	**3.7**	**46**	**21.3**	**216**	**100.0**	
Age									
25 to 39	60	64.5	3	3.2	30	32.2	93	100.0	*p*(1) = 0.009^*∗*^
40 to 59	76	81.7	5	5.4	12	12.9	93	100.0	
60 to 80	26	86.6	—	—	4	13.3	30	100.0	
Sex									
Male	35	77.8	2	4.4	8	17.8	45	100.0	*p*(1) = 0.955
Female	127	74.3	6	3.5	38	22.2	171	100.0	

^*∗*^Significant association at 5.0%.

(1) Through the Fisher exact test.

(2) Through Pearson's Chi-square test.

**Table 8:** Evaluation of the type of elongated styloid process on the left side according to age and sex.

	Type of elongation								*p* values
	Elongated		Pseudoarticulated		Segmented		Total		
	*n*	%	*n*	%	*n*	%	*n*	%	
**Total**	**54**	**1.3**	**8**	**3.7**	**54**	**25.0**	**216**	**100.0**	
Age									
25 to 39	5	9.1	3	3.2	35	37.6	93	100.0	*p*(1) = 0.002^*∗*^
40 to 59	5	0.6	5	5.4	13	14.0	93	100.0	
60 to 80	4	0.0	—	—	6	20.0	30	100.0	
Sex									
Male	2	1.1	2	4.4	11	24.4	45	100.0	*p*(1) = 0.955
Female	22	13	6	3.5	43	25.1	171	100.0	

^*∗*^Significant association at 5.0%.

(1) Through the Fisher exact test.

(2) Through Pearson's Chi-square test.

**Table 9:** Evaluation of calcification pattern of the second type of elongation on the right side.

Type of elongation (right side)	Calcified		Almost calcified		Calcified outline		Total		*p* values
	*n*	%	*n*	%	*n*	%	*n*	%	
**Total**	**24**	**11.1**	** 135**	** 62.5**	**57**	**26.4**	**216**	**100.0**	
Elongated	22	13.6	104	64.2	36	22.2	162	100.0	*p*(1) = 0.036^*∗*^
Pseudoarticulated	1	12.5	4	50.0	3	37.5	8	100.0	
Segmented	1	2.2	27	58.7	18	39.1	46	100.0	

^*∗*^Significant association at 5.0%.

(1) Through the Fisher exact test.

**Table 10:** Evaluation of calcification pattern of the second type of elongation on the left side.

Type of elongation (left side)	Calcified		Almost calcified		Calcified outline		Total		*p* values
	*n*	%	*n*	%	*n*	%	*n*	%	
**Total**	**23**	**10.6**	** 135**	** 62.5**	**58**	**26.9**	**216**	**100.0**	
Elongated	20	13.0	100	64.9	34	22.1	154	100.0	*p*(1) < 0.0001^*∗*^
Pseudoarticulated	3	37.5	3	37.5	2	25.0	8	100.0	
Segmented	—	—	32	59.3	22	40.7	54	100.0	

^*∗*^Significant association at 5.0%.

(1) Through the Fisher exact test.

## References

[1] Eagle W. W. (1958). Elongated styloid process: symptoms and treatment. *Archives of Otolaryngology*.

[2] Steinmann E. P. (1970). A new light on the pathogenesis of the styloid syndrome. *Archives of Otolaryngology*.

[3] Camarda A. J., Deschamps C., Forest D. (1989). II. Stylohyoid chain ossification: a discussion of etiology. *Oral Surgery, Oral Medicine, Oral Pathology*.

[4] İlgüy D., İlgüy M., FIşekçioğlu E., Dölekoğlu S. (2013). Assessment of the stylohyoid complex with cone beam computed tomography. *Iranian Journal of Radiology*.

[5] Tiago R. S. L., Marques Filho M. F., Santos Maia C. A., Souza Santos O. F. (2002). Eagle's syndrome: surgical treatment evaluation. *Revista Brasileira de Otorrinolaringologia*.

[6] Guzzo F. A. V., Macedo J. A. G. C., Barros R. S., Almeida D. C. (2006). Eagle's syndrome: a case report. *Revista Paraense de Medicina*.

[7] Langlais R. P., Miles D. A., Van Dis M. L. (1986). Elongated and mineralized stylohyoid ligament complex: a proposed classification and report of a case of Eagle's syndrome. *Oral Surgery, Oral Medicine, Oral Pathology*.

[8] Lima Júnior J. L., Rocha J. F., Ribeiro E. D., Costa V. S., Sousa E. M. (2007). Eagle's syndrome: a review of the literature. *Acta Odontológica Venezolana*.

[9] Ilgüy M., Ilgüy D., Güler N., Bayirli G. (2005). Incidence of the type and calcification patterns in patients with elongated styloid process. *The Journal of International Medical Research*.

[10] Guimarães S. M. R., Carvalho A. C. P., Guimarães J. P., Gomes M. B., Cardoso M. D. M. M., Reis H. N. (2006). Prevalence of morphological alterations of the styloid process in patients with temporomandibular joint disorder. *Radiologia Brasileira*.

[11] Guimarães A. G. P., Cury S. E. V., Silva M. B. F., Junqueira J. L. C., Torres S. C. M. (2010). Prevalence of elongated styloid process and/or ossified stylohyoid ligament in panoramic radiographs. *Revista Gaúcha de Odontologia*.

[12] More C. B., Asrani M. K. (2010). Evaluation of the styloid process on digital panoramic radiographs. *Indian Journal of Radiology and Imaging*.

[13] Ferrario V. F., Sigurta D., Daddona A. (1990). Calcification of the stylohyoid ligament: incidence and morphoquantitative evaluations. *Oral Surgery, Oral Medicine, Oral Pathology*.

[14] Tavares H., Freitas C. F. (2007). Prevalence of the elongated styloid process of temporal bone and calcification of the stylohyoid ligament by panoramic radiography. *Revista de Odontologia da Universidade da Cidade de São Paulo*.

[15] Lopes M. R. (2010). *Images of occurrence suggestive of styloid process of forming in panoramic radiographs [Dissertation (Graduate)]*.

[16] Keur J. J., Campbell J. P. S., Mccarthy J. F., Ralph W. J. (1986). The clinical significance of the elongated styloid process. *Oral Surgery, Oral Medicine, Oral Pathology*.

[17] Ruprecht A., Sastry K. A., Gerard P., Mohammad A. R. (1988). Variation in the ossification of the stylohyoid process and ligament. *Dentomaxillofacial Radiology*.

[18] Watanabe P. C. A., Campos M., Pardini L. C. (1998). Syndrome of the elongated styloid process (Eagle's Syndrome). *Revista da Associação Paulista de Cirurgiões Dentistas*.

[19] Gonτales E. S., Nary FIlho H., Alvarez L. C., Oliveira C. M., Stanghini V. (2003). Eagle's syndrome: radiographic study of the incidence of elongated styloid process. *Revista Salusvita*.

[20] Pinto P. R. O., Vieira G. L., Menezes L. M., Rizzatto S. M. D., Brücker M. R. (2008). Evaluation of the styloid process in subjects with class III malocclusion. *Revista Odonto Ciência*.

[21] de Paula M. V. Q., Carraretto F. G. (2008). Prevalence of elongation of the styloid process in patients with temporomandibular disorders. *Revista da Imagem*.

[22] Monsour P. A., Young W. G. (1986). Variability of the styloid process and stylohyoid ligament in panoramic radiographs. *Oral Surgery, Oral Medicine, Oral Pathology*.

[23] Eagle W. W. (1937). Elongated styloid process. *Archieves of Otolaryngology*.

[24] Prasad K. C., Kamath M. P., Reddy K. J. M., Raju K., Agarwal S. (2002). Elongated styloid process (Eagle's syndrome): a clinical study. *Journal of Oral and Maxillofacial Surgery*.

[25] Correll R. W., Jensen J. L., Taylor J. B., Rhyne R. R. (1979). Mineralization of the stylohyoid-stylomandibular ligament complex. A radiographic incidence study. *Oral Surgery, Oral Medicine, Oral Pathology*.

[26] Erol B. (1996). Radiological assessment of elongated styloid process and ossified stylohyoid ligament. *Journal of Marmara University Dental Faculty*.

